# Tourniquet effect on cement penetration in total knee arthroplasty: A systematic review and meta‐analysis

**DOI:** 10.1002/jeo2.70380

**Published:** 2025-11-14

**Authors:** Alireza Mirahmadi, Ava Parvandi, Mahdi Mohammaditabar, Donya Rezazadeh Eidgahi, Amirsina Shaker Dorabad, Shayan Amiri, Hamed Tayyebi, Mengnai Li, Ara Nazarian

**Affiliations:** ^1^ Musculoskeletal Translational Innovation Initiative, Carl J. Shapiro Department of Orthopaedic Surgery, Beth Israel Deaconess Medical Center Harvard Medical School Boston Massachusetts USA; ^2^ Bone and Joint Reconstruction Research Center, Department of Orthopedics, School of Medicine Iran University of Medical Sciences Tehran Iran; ^3^ Bone Joint and Related Tissues Research Center, Akhtar Orthopedic Hospital Shahid Beheshti University of Medical Sciences Tehran Iran; ^4^ University of Arizona College of Medicine Phoenix Arizona USA; ^5^ Carl J. Shapiro Department of Orthopaedic Surgery Beth Israel Deaconess Medical Center Boston Massachusetts USA; ^6^ Department of Orthopaedic Surgery Yerevan State Medical University Yerevan Armenia

**Keywords:** bone cement, cement mantle thickness, cement penetration, knee zone, meta‐analysis, total knee arthroplasty, tourniquet

## Abstract

**Purpose:**

Total knee arthroplasty (TKA) is a standard orthopaedic procedure for severe knee arthritis, often resulting in high patient satisfaction. However, complications, such as aseptic loosening, remain a significant concern, some thought to be linked to insufficient cement penetration. Using a tourniquet during surgery to improve cement penetration is a topic of debate, with evidence regarding its mixed effectiveness. This review aims to evaluate the impact of tourniquet application on cement penetration, TKA outcomes and related complications.

**Methods:**

A meta‐analysis adhering to the Preferred Reporting Items for Systematic Reviews and Meta‐Analyses guidelines was conducted, analyzing comparative studies from PubMed, Scopus, Web of Science and Embase. Eligibility criteria focused on studies assessing tourniquet effects on cement penetration, complications and other surgical outcomes. Data extraction and quality assessment followed standardized protocols. Statistical analyses employed a random‐effects model to account for heterogeneity, including sensitivity analyses and publication bias assessments.

**Results:**

The meta‐analysis included 16 studies encompassing 1516 observations. Tourniquet use significantly increased cement penetration in average and cumulative analysis (*p* value = 0.045 and 0.005, respectively). Tourniquet pressure‐based subgroup analysis did not show statistically significant differences in cement penetration between groups. In secondary outcomes, a 52% reduction in blood transfusion likelihood was observed in the tourniquet group—no significant differences in haemoglobin levels (standardized mean difference [SMD] = 0.0; *p* = 1). No differences were noted in surgical time (SMD = −0.152; *p* = 0.26) or postoperative pain (visual analogue scale scores; *p* = 0.184).

**Conclusions:**

Tourniquet usage enhanced cement penetration but did not significantly affect surgical duration and pain; instead, it reduced blood transfusion rates. However, variability in surgical techniques and methodologies among included studies has contributed to the results. Future research must use standardized methodologies to resolve inconsistencies and confirm these results.

**Levels of Evidence:**

Level II.

**Abbreviations**
AP: anteroposterior
BMI: body mass index
CI: confidence interval
DVT: deep vein thrombosis
Hb: haemoglobin
NRSI: non‐randomized studies of intervention
PRISMA: Preferred reporting items for systematic reviews and meta‐analyses
RCT: randomized clinical trial
SD: standard deviation
SMD: standardized mean difference
TKA: total knee arthroplasty
VAS: visual analogue scale

## INTRODUCTION

Total knee arthroplasty (TKA) is a routine and highly effective surgical intervention for managing severe knee arthritis, with patient satisfaction rates reported to reach as high as 91% [32]. Despite its success, one of the most significant complications following TKA is the need for revision surgery, often due to the loosening of the tibial component [48]. Recent studies indicate that approximately 2%–5% of revision TKAs are attributed to prosthetic loosening, which can significantly impact patient outcomes and healthcare costs [32]. Aseptic loosening, a common cause of prosthesis failure, can result from various factors, including wear and tear, implant malalignment, poor prosthetic design and inadequate bonding at the bone–cement interface [6, 24].

The penetration of the cement mantle and the strength of the cement–bone interface are crucial determinants of primary TKA success. Since bone cement lacks inherent adhesive properties, adequate penetration into the trabecular bone is essential for achieving mechanical interlock and ensuring implant stability [8, 12, 23, 48]. Furthermore, increasing the cement penetration depth has improved implant longevity and strength [8, 34, 45]. One intraoperative factor that may influence cementation quality is the use of a tourniquet. By creating a bloodless surgical field, tourniquet application is thought to enhance bone surface preparation, reduce blood and fat interference, and improve cement penetration [46, 50].

The impact of tourniquet use on cement mantle penetration is still debated in the literature. While some studies report that using a tourniquet increases cement penetration, potentially improving the stability of the prosthesis [18, 21, 46, 51], other studies have found no significant effect on cement mantle thickness [22, 26, 43, 53, 57]. Given these conflicting findings, it is uncertain whether using a tourniquet during TKA enhances the thickness of the cement mantle around the tibial component, thereby improving the prosthesis's stability and durability.

To address this uncertainty, we conducted a meta‐analysis of randomized controlled trials (RCTs) and non‐randomized studies of interventions (NRSIs) that evaluated the impact of tourniquet use on the thickness of the bone cement around the tibial prosthesis. This study aims to clarify whether tourniquet application during TKA contributes to improved cement penetration, which may influence prosthetic fixation.

## METHODS

This systematic review and meta‐analysis adhered to the Preferred Reporting Items for Systematic Reviews and Meta‐Analyses (PRISMA) guidelines and was registered in the PROSPERO database under the registration code (CRD42024627837) [44]. This study evaluated whether tourniquet application during TKA enhances prosthetic stability through improved cementing. We conducted an extensive literature search from the start of indexing each database through February 2025, including MEDLINE (PubMed), Web of Science, Embase and Scopus. The search strategy combined Medical Subject Headings (MeSH) and free‐text terms such as ‘total knee arthroplasty’ OR ‘total knee replacement’ OR ‘TKA’ AND ‘tourniquet’ using Boolean operators. The complete search strategy for all databases is available in Appendix A.

### Eligibility criteria

This study seeks to comprehensively evaluate the impact of tourniquet use on cement‐related critical clinical and procedural parameters in TKA. The search strategy identified and included all RCTs and NRSIs that compared the effects of tourniquet use versus no tourniquet use on tibial bone cement penetration in primary TKA. Studies were included if they reported P: Patients underwent TKA, I: using a tourniquet, C: Not using a tourniquet, Outcomes: cement penetration rates and related variables. Exclusion criteria applied to studies that did not feature the specified comparative groups, focused on revision TKA procedures, lacked sufficient data for analysis, or were classified as meta‐analyses, reviews (systematic, narrative, scoping, comprehensive), letters to the editor, conference abstracts, comments, case series and case reports, non‐comparative cohorts, animal, in vitro and cadaveric studies. These criteria were designed to ensure the inclusion of high‐quality evidence for assessing the impact of intraoperative tourniquet use in TKA.

### Study selection

**Figure 1:**
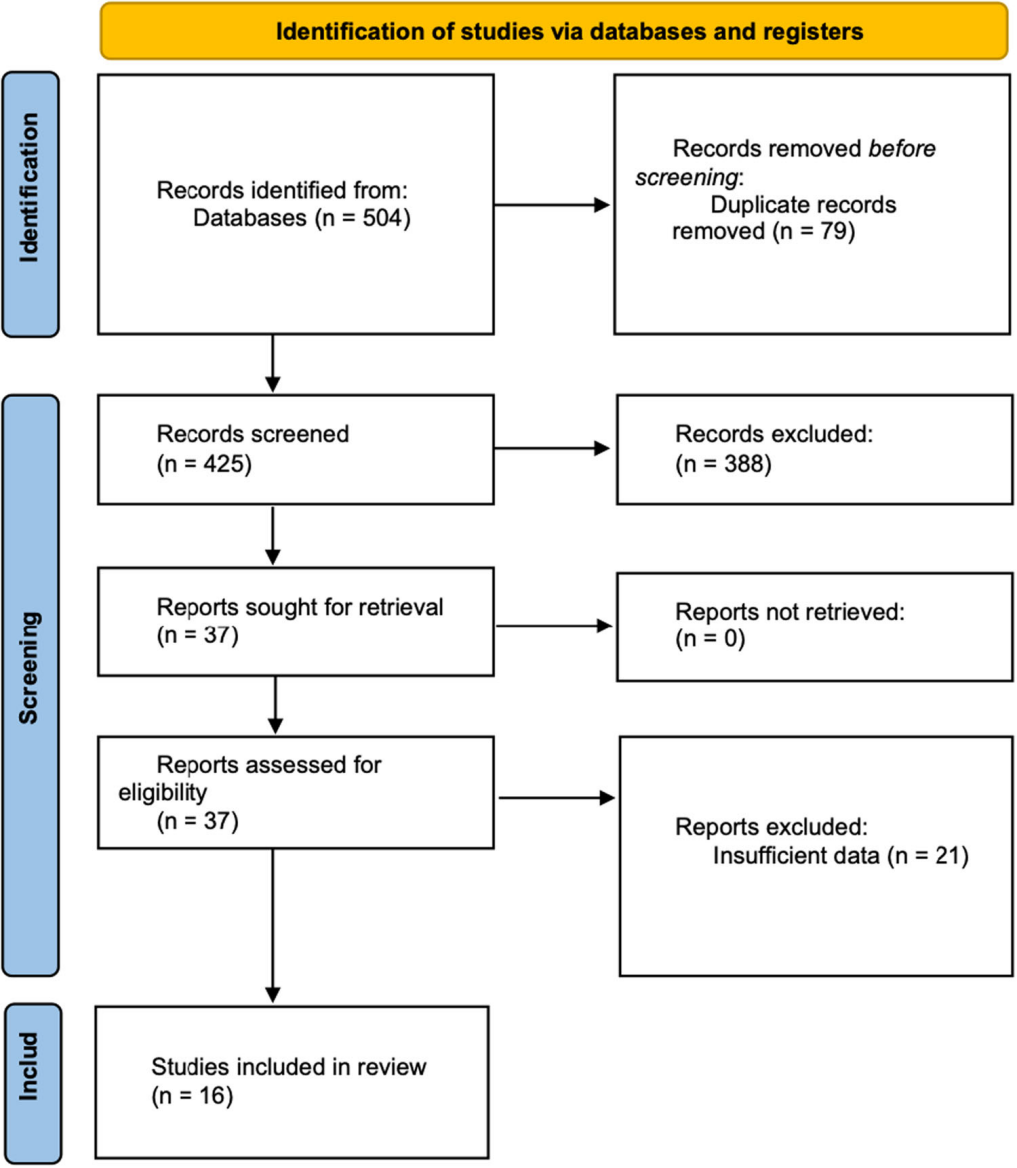
PRISMA flow chart for Study selection. PRISMA, preferred reporting items for systematic reviews and meta‐analyses.

Two independent reviewers (AP and DRD) screened the titles and abstracts of the papers. Subsequently, the full texts of the studies were gathered and assessed by two independent reviewers (AP and DRD) for inclusion. Disagreements in each step were resolved through discussion or guidance from a third, more experienced reviewer (SA). The PRISMA flow diagram depicts the study selection process (Figure 1).

### Data extraction

Three reviewers (AP, DRD and ASD) independently extracted data using a standardized form. Extracted data included authors, sample size, year of publication, intervention, assessment tool, outcomes, descriptions of control and intervention arms, and total number of participants in each trial. In this study, we aim to compare the outcomes of TKA performed with and without a tourniquet. Patients who underwent TKA will be divided into two groups: one undergoing TKA with the application of a tourniquet and the other without its use. Qualified studies' events, means and standard deviations (SDs) were used for meta‐analysis.

**Figure 2:**
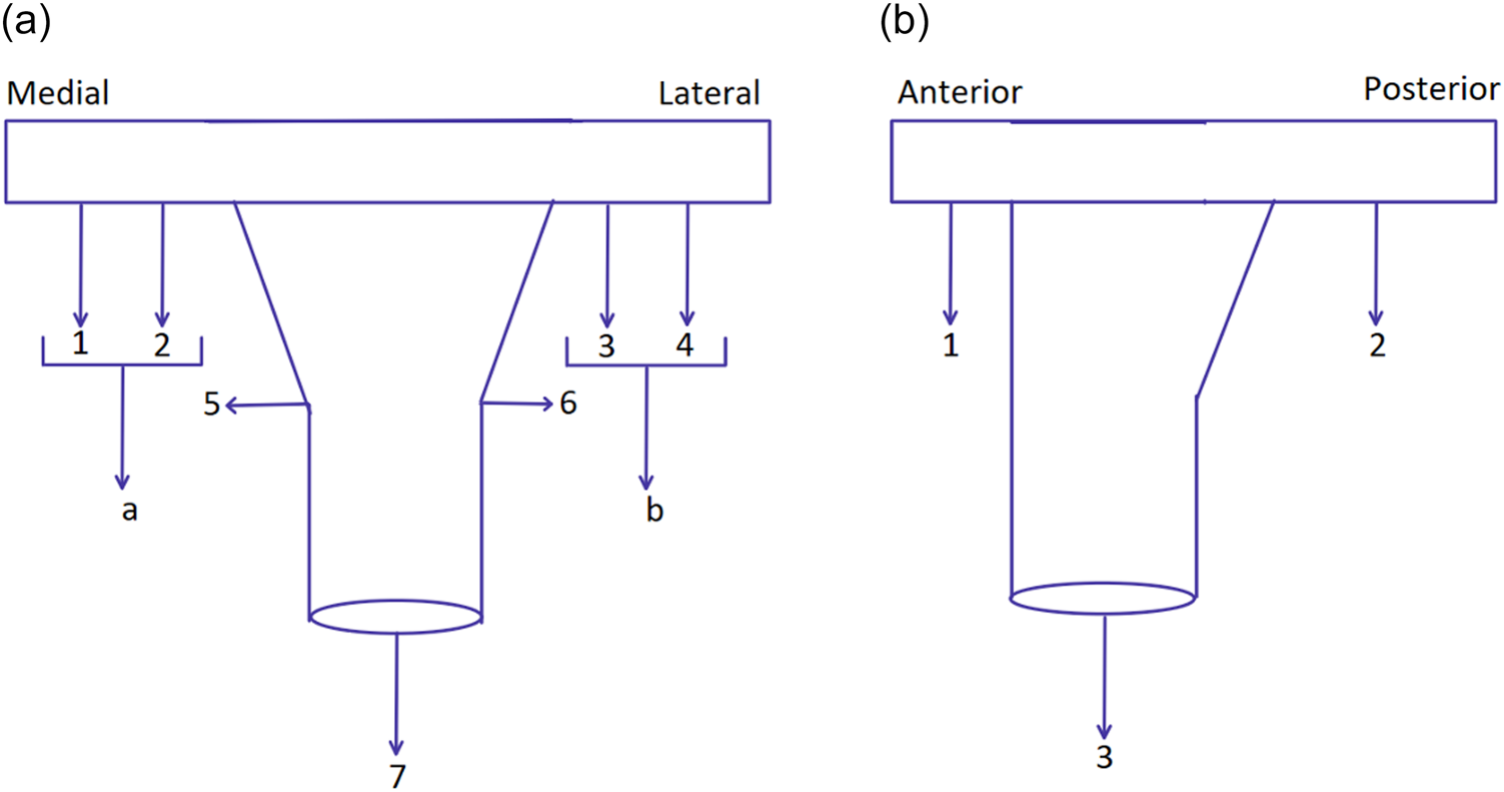
The figure illustrates the distribution of the tibial cement mantle in various zones based on the Knee Society Score classification in both anteroposterior (AP) and lateral views. In the AP view, the medial side is divided into Zones 1 and 2, while the lateral side comprises Zones 3 and 4. When the article refers to the medial side without specifying Zone 1 or 2, it is classified as Zone a. Similarly, if only the lateral side is mentioned without specifying Zone 3 or 4, it is classified as Zone b. Additionally, the AP view includes Zones 5–7. In the lateral view, the classification consists of Zones 1–3. This approach ensures a standardized interpretation when specific zone details are not provided.

Data were extracted based on study characteristics, population details and intervention parameters. Study characteristics included information on the authors, study type and year of publication. Population details comprised gender and body mass index (BMI). Intervention parameters involved tourniquet pressure, duration, brand of bone cement and cementing technique. The primary outcome was the thickness of tibial cement mantle penetration, measured in specific zones on the anteroposterior (AP) and lateral views according to the Knee Society Scoring System. Tibial AP Zones 1–4 (Figure 2) represent the medial and lateral inferior surfaces of the tibial baseplate, while lateral Zones 1 and 2 correspond to the anterior and posterior distal surfaces. Cement penetration depth was evaluated at one third, two thirds or one half marks. The cumulative penetration depth was calculated as the sum of all measurements using the Picture Archiving and Communication System.

Secondary outcomes included the duration of surgery, changes in haemoglobin (Hb) levels, transfusion rates, postoperative visual analogue scale (VAS) pain scores, and complications such as infections and deep vein thrombosis (DVT). These outcomes provided additional insights into the broader clinical impact of tourniquet use in TKA.

### Quality assessment

**Figure 3:**
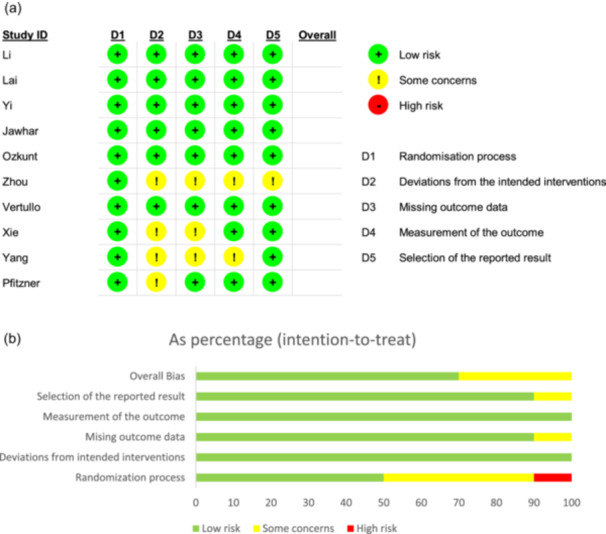
Risk of bias summary for RCT articles. Review authors' judgements about each risk of bias item for each included study (a) a summary bar chart shows the risk of bias assessments across domains for included RCTs and NRSIs (b). NRSI, non‐randomized studies of intervention; RCT, randomized clinical trial.

Two researchers (AP and ASD) independently investigated study quality using the Revised Cochrane risk of bias tool for randomized trials (RoB2) (Figure 3) and the Newcastle–Ottawa Scale for NRSIs (Table 1). This scale includes eight questions about selection, comparability, and outcomes. Any disagreements were resolved through discussion or guidance from a third, more experienced reviewer.

**Table 1:** Risk of bias summary for NRSI articles. Review authors' judgements about each risk of bias item for each included study.

	Selection	Comparability	Exposure	Total
Kizilkurt	★★★	★★	★★	7
Hegde	★★★	★★	★★	7
Herndon	★★★	★★	★★	7
Gao	★★★	★★	★★	7
Gapinski	★★★	★★	★★	7
Touzopoulos	★★★	★★	★★	7

Abbreviation: NRSI, non‐randomized studies of intervention.

### Statistical analysis

Meta‐analysis and statistical analysis were performed using R. We used standardized mean differences (SMDs) as the summary statistic for continuous data by gathering the tourniquet use and control groups' mean (SD) and sample size (*n*). All data were analyzed at a single point at the end of the trial—the effect of using a tourniquet on bone cement penetration and other complications. Between‐study heterogeneity was investigated using the *I* ^2^ test since an *I* ^2^ of around 25%, 50% and 75% is considered low, moderate and high levels of heterogeneity, respectively. The random‐effects model was supposed to be calculated. Publication bias was assessed using Egger's test and a funnel plot. We further explored each study's influence through the graphical display of study heterogeneity analysis and three unsupervised learning algorithms to detect potential outliers and make our analysis more homogeneous. Egger's test evaluated asymmetry; *p* < 0.05 was set as the significance level.

## RESULTS

### Search results

The literature search strategy and selection process are presented in Figure 1. After removing duplicates, 425 studies were initially identified. Screening of titles and abstracts resulted in the exclusion of 388 studies deemed irrelevant to the clinical focus. A subsequent review of 37 full‐text articles led to the exclusion of 21 additional studies due to the absence of comparative groups or insufficient outcome data. Ultimately, 16 articles from 2014 to 2024 were deemed suitable for inclusion, comprising 10 RCTs [26, 31, 35, 43, 46, 53, 57, 58, 59, 61] and 6 NRSIs [17, 18, 21, 22, 28, 51], collectively assessing 1516 patients. The initial analysis was based on the concept that using a tourniquet in patients undergoing TKA can cause any changes in bone cement penetration (Table 2).

**Table 2:** Demographics.

Author/year	Journal	Type of study	Number of patients use/control	Gender M, F use/control	Age (years) use/control	BMI use/control	Surgery duration use/control	Tourniquet pressure (mmHg)	Tourniquet time (min)	Bone cement technique/brand	Complications (use)	Complications (control)
Li et al., 2024 [35]	Joint Diseases and Related Surgery	RCT	94/89	19,75/24,65	68.13 ± 6.34/67.45 ± 6.54	26.37 ± 3.74/27.01 ± 3.23	79.90 ± 8.19/81.65 ± 8.36	300	Full‐time (continuous)	NM	NM	NM
Kizilkurt et al., 2022 (with TXA) [28]	European Journal of Orthopaedic Surgery & Traumatology	RCS	28/24	8,20/9,15	63.2(55–66)/65.7(61–69)	NM/NM	NM/NM	300	Full‐time (continuous)	Fourth generation/PMMA	NM	NM
Kizilkurt et al., 2022 (without TXA) [28]	European Journal of Orthopaedic Surgery & Traumatology	RCS	29/38	11,18/14,24	65.1(59–71)/67.2(62–73)	NM/NM	NM/NM	300	Full‐time (continuous)	Fourth generation/PMMA	NM	NM
Lai et al., 2022 [31]	Journal of Orthopaedic Surgery and Research	RCT	14/14	4,10/3,11	64.00 ± 6.56/65.57 ± 7.70	27.76 ± 3.266/27.40 ± 3.476	95 (80–101.25)/107 (93.75–135)	240	Full‐time (continuous)	NM	Tension blisters: 4 Ecchymosis: 2 Numbness of lower limbs: 1 Superficial infection: 0 Deep vein thrombosis (DVT): 2	Tension blisters: 1 Ecchymosis: 1 Numbness of lower limbs: 0 Superficial infection: 0 DVT: 1
Hegde et al., 2021 [21]	The Journal of Arthroplasty	RCS	61/61	37,24/37,24	63.64 ± 7.02/63.66 ± 7.26	30.43 ± 4.81/29.48 ± 5.15	NM/NM	250	Full‐time (continuous) (>30)	Fourth generation/Simplex P (Stryker Orthopedics Inc)	None	2 aseptic tibial loosening requiring revision
Yi et al., 2021 [59]	BMC Musculoskeletal Disorders	RCT	50/50	7,43/8,42	68.44 ± 6.80/68.00 ± 7.11	26.13 ± 2.63/25.34 ± 3.61	76.60 ± 8.72/79.30 ± 8.21	100 ↑ sBP	61.00 ± 6.55	Third generation/Smartest GMV Endurance, DePuy, Blackpool, England	1 wound superficial cellulitis 2 calf muscular venous thrombosis	NA
Herndon et al., 2020 [22]	Knee Surgery, Sports Traumatology, Arthroscopy	RCS	70/70	28,42/26,44	67.0 ± 9.2/67.5 ± 8.3	NM/NM	108.8 ± 20.3/98.7 ± 22.3	250	Full‐time (continuous)	Third generation/Simplex (Stryker, Kalamazoo, MI, USA) with Tobramycin	NM	NM
Gao et al., 2019 [17]	Chinese Journal of Bone and Joint Injury	RCS	29/29	7,22/9,10	62.3/63.7	25.72/26.13	NA	280–350	Full‐time (continuous)	Third generation	NA	NA
Gapinski et al., 2019 [18]	The Journal of Arthroplasty	RCs	90/79	20,70/18,61	67.4 ± 9.7/66.9 ± 7.9	33.6/35	NM/NM	NA	Full‐time (continuous)	Third generation/Medium viscosity PMMA with low‐dose antibiotics	NM	NM
Jawhar et al., 2019 [26]	Knee Surgery, Sports Traumatology, Arthroscopy	RCT	43/43	16,27/16.27	70 ± 6.8/71 ± 6.8	31.9 ± 5.7/31.9 ± 5.7	79 ± 23/85 ± 20	360 ± 20	82.3 ± 20	Third generation/SmartSet Bone cement, DePuySynthes, Warsaw, IN, USA	1 DVT + 1 revision surgery due to surgical site infection	1 delayed wound healing without a need for revision surgery
Touzopoulos et al., 2019 [51]	European Journal of Orthopaedic Surgery & Traumatology	RCS	50/50	42,8/42,8	70.73 ± 6.56/69.92 ± 6.89	31.04 ± 5.43/31.32 ± 3.95	NM/NM	350	Full‐time (continuous)	Fourth generation/Palacos R + G®, Heraeus, Hanau, Germany	None	None
Ozkunt et al., 2018 [43]	Medicine	RCT	24/25	NM/NM	NM/NM	NM/NM	NM/NM	NM	Limited or transient tourniquet	Third generation/low‐viscosity PMMA	None	None
Zhou et al., 2018 [61]	Chinese Journal of Rural Medicine and Pharmacy	RCT	49/49	26,23/24,25	62.7/62.5	26.1/26.4	NA	100 ↑ sBP	Full‐time (continuous)	Third generation	Superficial infection: 2 MCVT: 6	Superficial infection: 2 MCVT: 4
Vertullo et al., 2017 [53]	Journal of Orthopaedic Surgery	RCT	20/20	10,10/11,9	67.85 ± 6.91/65.65 ± 8.54	30.43 ± 5.07/31 ± 5.31	NM/NM	300	Full‐time (continuous)	Third generation/Vacuum‐mixed Palacos RþG PMMA (Zimmer)	None	None
Xie et al., 2017 [57]	Chinese Journal of Orthopaedics	RCT	45/45	6,39/11,34	66.2/66.1	26.1/25.9	NA	100 ↑ sBP	Full‐time (continuous)	Third generation	NA	NA
Yang et al., n.d. [58]	Chongqing Medicine	RCT	41/41	8,33/7,34	62.8/66.3	33.6/35	70.4 ± 16.6/76.4 ± 11.8	100 ↑ sBP	Full‐time (continuous)	Third generation/Smartest	DVT: 0 MCVT: 15	DVT: 0 MCVT: 6
Pfitzner et al., 2014 [46]	Knee Surgery, Sports Traumatology, Arthroscopy	RCT	45/45	21,24/11,34	69.3 (47–85)/70.5 (50–90)	27.8 (18.5–38.1)/26 (18.5–33.9)	NM	350	NM	Fourth generation/Palacos R®, Heraeus, Hanau, Germany	NM	NM

Abbreviations: MCVT, major cardiovascular thrombotic events; RCS, retrospective cohort study; RCT, randomized clinical trial; sBP, systolic blood pressure.

### The effect of using a tourniquet on bone cement penetration

**Figure 4:**
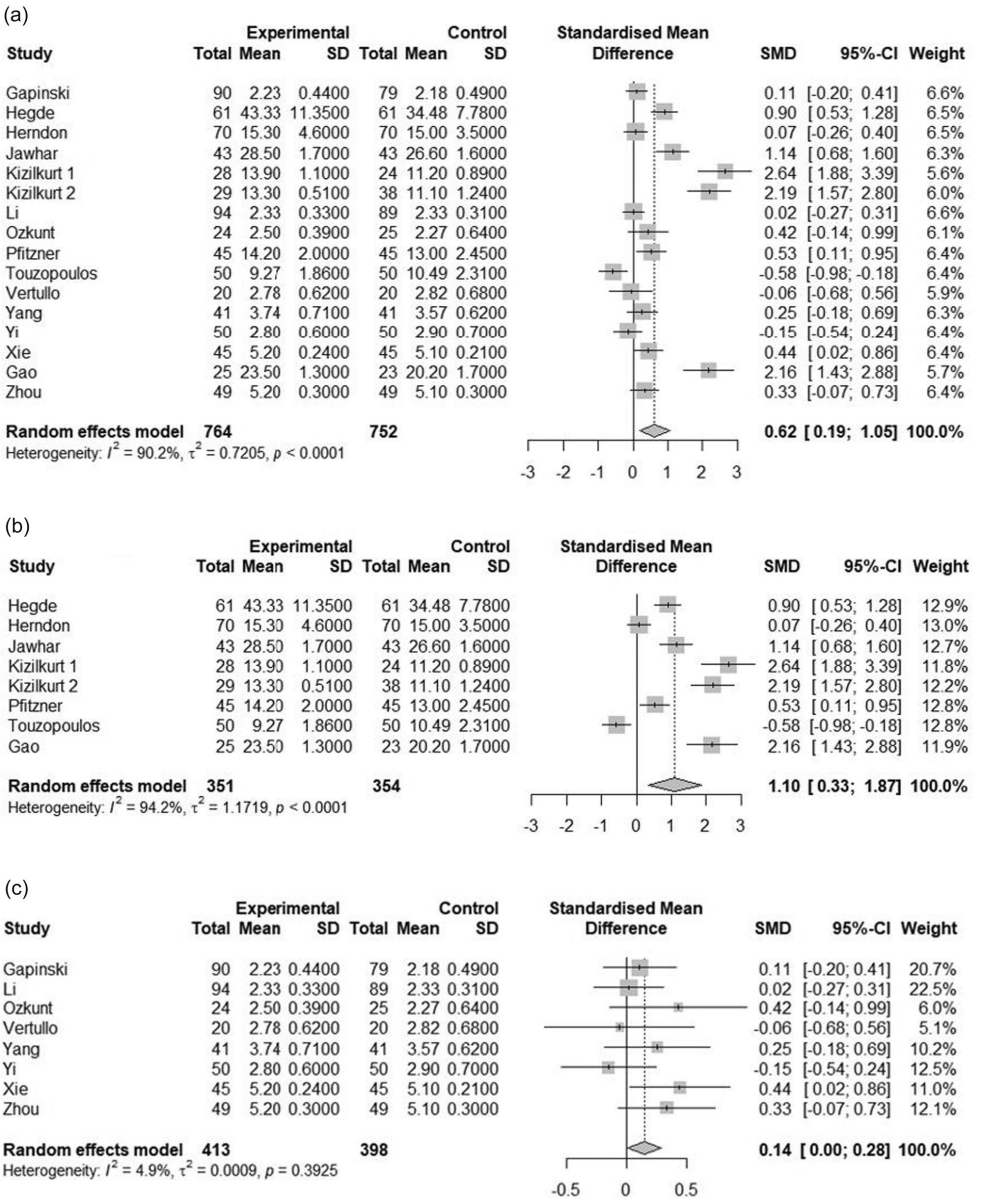
Forest plot of cement penetration analysis in TKA patients in total (a), cumulated (b) and average (c) penetration. CI, confidence interval; SD, standard deviation; SMD, standardized mean difference; TKA, total knee arthroplasty.

The meta‐analysis included 1516 observations (tourniquet = 764, control = 752) from 16 studies. This analysis showed that using a tourniquet can improve bone cement penetration on tibial components (SMD = 0.619, 95% confidence interval [CI] = [0.186–1.052], *p* value = 0.005) (Figure 4).

**Figure 5:**
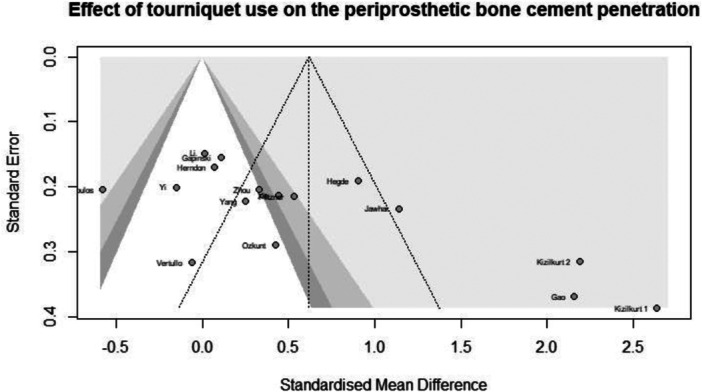
Funnel plot of cement penetration analysis in TKA patients (in total). TKA, total knee arthroplasty.

Sensitivity analysis conducted using the leave‐one‐out method showed that all the pooled estimates after one survey at a time were still within the 95% CI of the overall estimate. Egger's linear regression test indicated significant asymmetry (*t* = 3.38, df = 14, *p* = 0.005), suggesting potential small‐study effects. The estimated bias was 7.986 (SE = 2.360, *τ* ^2^ = 6.014). The funnel plot and Egger's test results indicated the presence of funnel plot asymmetry (intercept = 7.98, 95% CI = [3.36–12.61], *p* value < 0.004) (Figure 5).

### The effect of using a tourniquet on bone cement penetration (average)

The meta‐analysis included 811 observations (tourniquet = 413, control = 398) from 8 studies. This analysis was only based on the articles that reported the average cement penetration. This analysis showed that using a tourniquet can improve tibial component‐related bone cement penetration (SMD = 0.144, 95% CI = [0.003–0.284], *p* value = 0.045) (Figure 4).

Sensitivity analysis conducted using the leave‐one‐out method showed that all the pooled estimates after one survey at a time were still within the 95% CI of the overall estimate.

### The effect of using a tourniquet on bone cement penetration (cumulative)

The meta‐analysis included 705 observations (tourniquet = 351, control = 354) from 8 studies. This analysis was only based on the articles that reported the cumulative cement penetration. This analysis showed that using a tourniquet can improve tibial component‐related bone cement penetration (SMD = 1.0991, 95% CI = [0.326–1.872], *p* value = 0.005) (Figure 4).

Sensitivity analysis conducted using the leave‐one‐out method showed that all the pooled estimates after one survey at a time were still within the 95% CI of the overall estimate. The funnel plot and Egger's test results indicated the presence of funnel plot asymmetry (Intercept = 11.98, 95% CI = [5.19–18.78], *p* value < 0.013).

**Figure 6:**
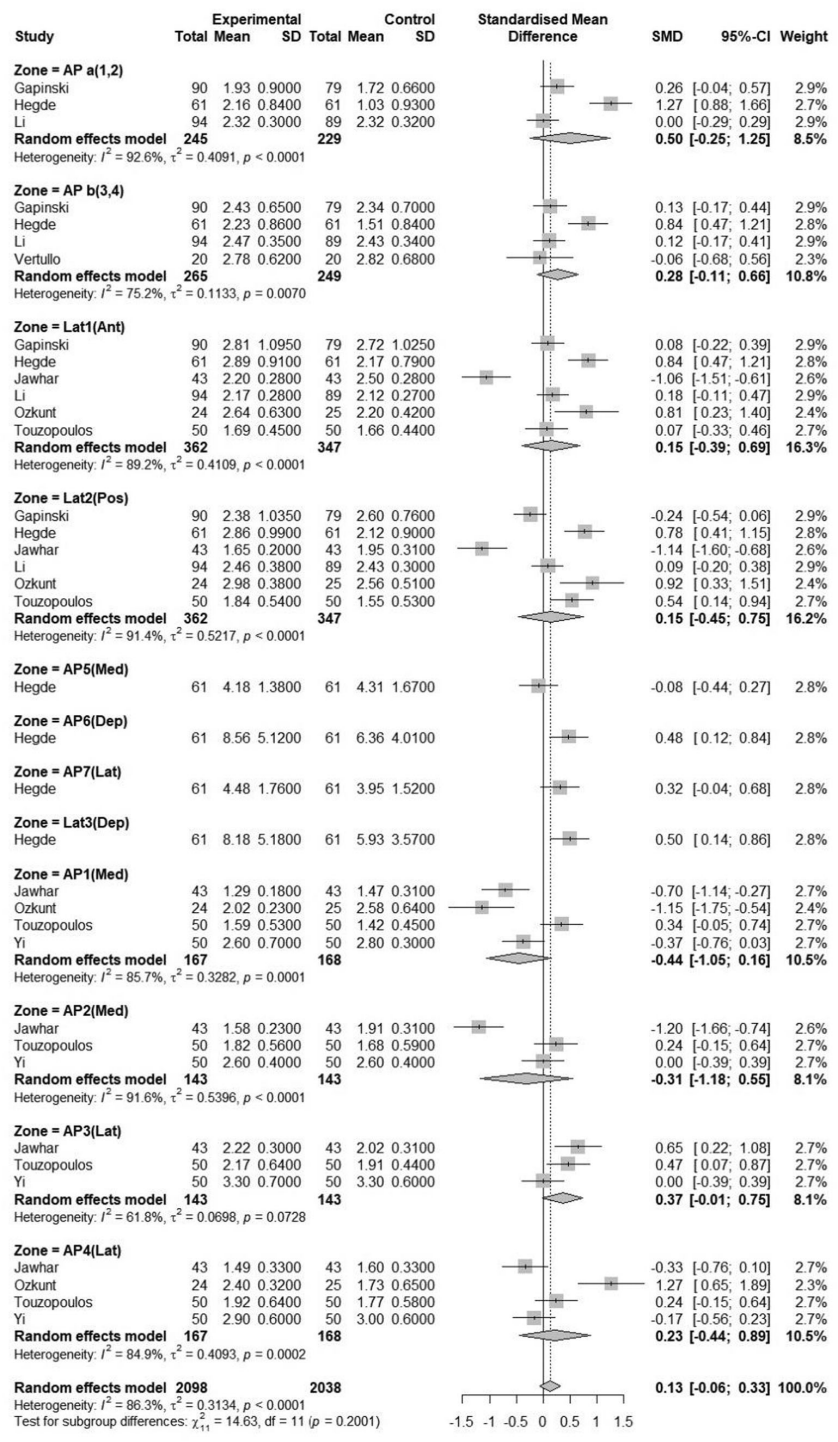
Forest plot of penetration in different zones (subgroup analysis) in TKA patients of eligible studies. CI, confidence interval; SD, standard deviation; SMD, standardized mean difference; TKA, total knee arthroplasty.

**Figure 7:**
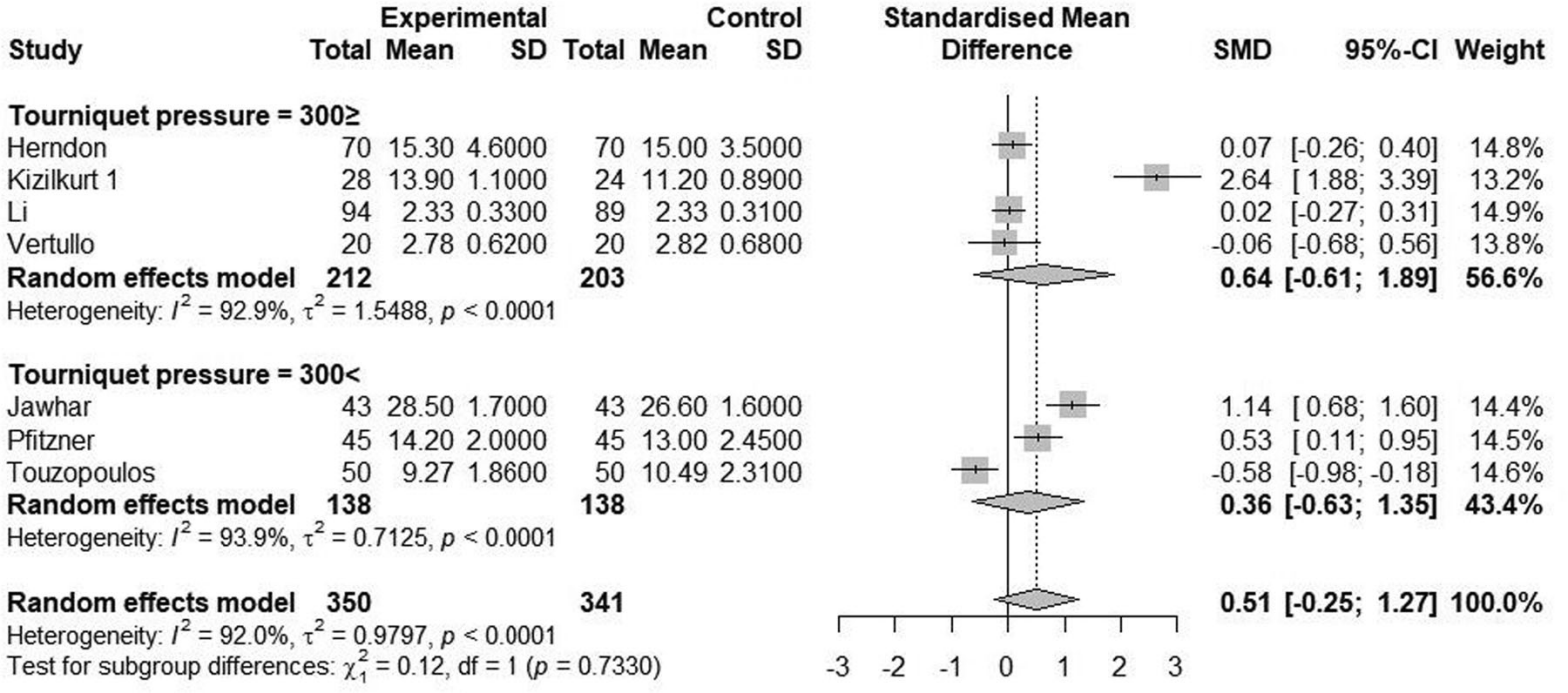
Forest plot of penetration in different tourniquet pressures (subgroup analysis) in TKA patients of eligible studies. CI, confidence interval; SD, standard deviation; SMD, standardized mean difference; TKA, total knee arthroplasty.

Subgroup analysis was conducted based on the pressure of the tourniquet (≤300 and >300 mmHg) and zones (Figures 6 and 7). Tourniquet pressure‐based subgroup analysis did not show statistically significant differences in cement penetration between groups. Zone‐based subgroup analysis revealed that certain zones, such as ‘AP a (1,2)’ (SMD = 0.50, 95% CI = [−0.25 to 1.25]), showed moderate effects, whereas others, such as ‘Lat 2(Pos)’ (SMD = 0.15, 95% CI = [−0.45 to 0.75]), demonstrated negligible or non‐significant effects. The heterogeneity within subgroups ranged from moderate to high (*I* ^2^ = 61.8%–92.6%), highlighting substantial differences in study design, population or intervention effects. While the findings suggest potential benefits of the experimental condition in some contexts, the high heterogeneity warrants cautious interpretation, requiring further investigation to explore the sources of variability.

**Figure 8:**
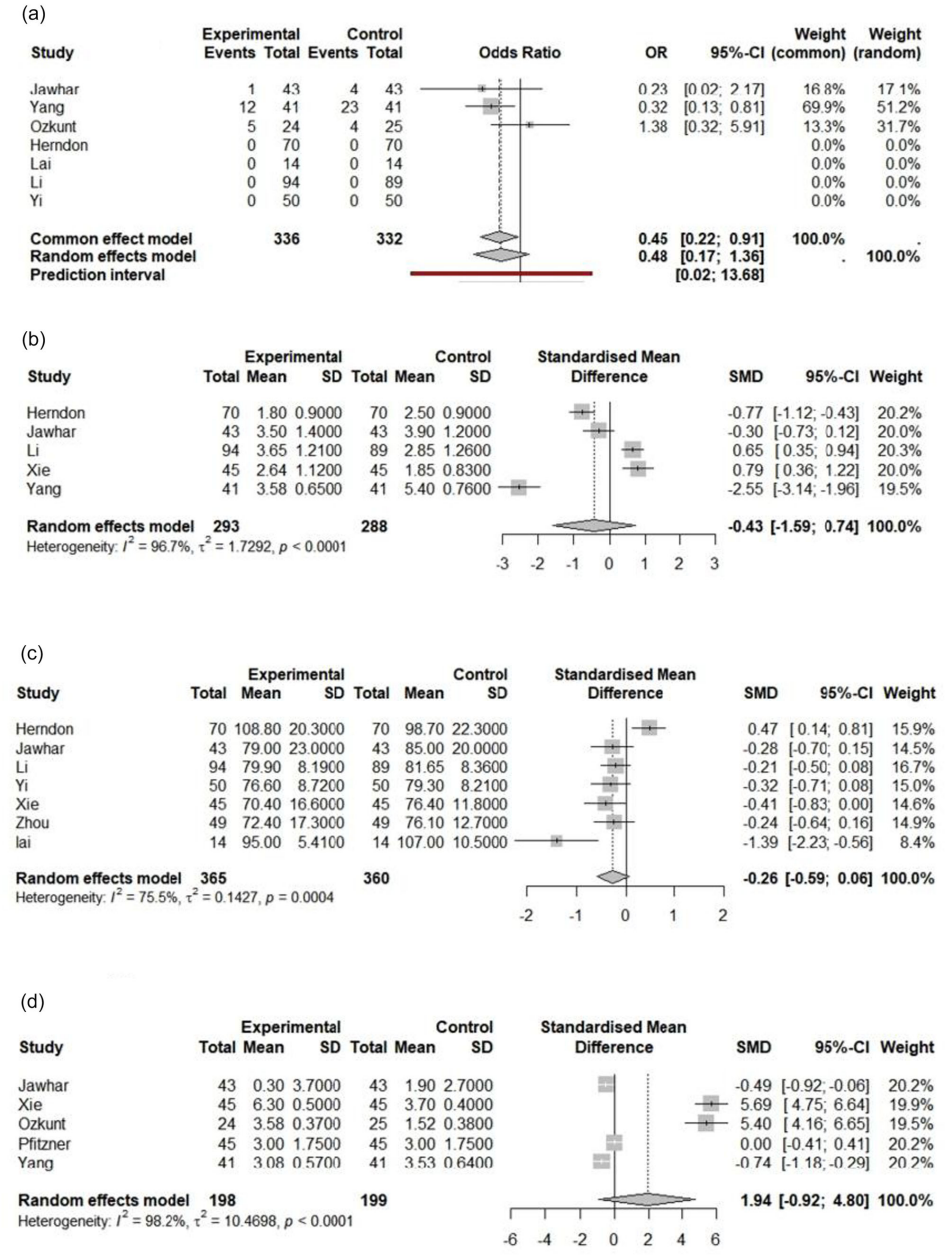
Forest plot of (a) blood transfusion rate analysis, (b) change in haemoglobin analysis, (c) operation duration analysis and (d) VAS score difference analysis in TKA patients of eligible studies. CI, confidence interval; SD, standard deviation; SMD, standardized mean difference; TKA, total knee arthroplasty; VAS, visual analogue scale.

Although the primary goal of this meta‐analysis was bone cement penetration, the following secondary outcome analyses were conducted (Figure 8).

### Blood transfusion rate

The meta‐analysis comparing blood transfusion rates between the experimental and control groups revealed a significant reduction in transfusion rates under the common effect model (OR = 0.45, 95% CI = [0.22–0.91]), while the random effects model did not show statistical significance (OR = 0.48, 95% CI = [0.17–1.36]). Individual study results varied, with some (e.g., Jawhar and Herndon) crossing the null value, indicating no significant difference. Moderate heterogeneity was observed (*I* ^2^ = 36, *p* = 0.210), suggesting some variability among studies that may not be due to chance.

### Change in Hb

The pooled effect size, calculated using a random‐effects model, was −0.45 (95% CI = [−1.594 to 0.743]; *p* = 0.476), indicating no statistically significant difference between the control and tourniquet groups. The test for heterogeneity was significant, with a high level of heterogeneity observed across studies (*I* ^2^ = 96.7%, [94.5%–98.1%]).

### Operation duration

The meta‐analysis included 725 observations (tourniquet = 365, control = 360) from 7 studies that showed no difference in operation duration between using a tourniquet in patients undergoing TKA compared to control (SMD = −0.261, 95% CI = [−0.586 to 0.063], *p* value = 0.115).

### VAS score difference

The meta‐analysis included 397 observations (tourniquet = 198, control = 199) from 5 studies that showed no difference in VAS score between using a tourniquet in patients undergoing TKA (SMD = 1.939, 95% CI = [−0.918 to 4.796], *p* value = 0.184).

## DISCUSSION

Our study evaluates tibial bone cement penetration across various zones on AP and lateral radiographic views in primary TKA performed with and without a tourniquet. The findings of the meta‐analysis indicate that using a tourniquet in TKA significantly influences cement penetration and increases cement mantle thickness but does not affect postoperative pain, surgery duration, or Hb levels. However, it reduces the likelihood of requiring a blood transfusion. The increase in cement penetration is predominantly observed in Zone 3 on the AP view. However, no significant increase in tibial cement mantle thickness was observed in Zones 1, 2 and 4 on the AP view and Zones 1 and 2 on the lateral view.

The initial fixation strength of the tibial component is widely recognized as a critical factor in ensuring the long‐term functionality of an implant. Aseptic loosening, a severe complication, typically occurs at the bone–cement interface [37]. Consequently, increasing the cement penetration thickness may be beneficial for establishing a robust cement–bone bond. This bond significantly enhances the implant's resistance to shear forces [25, 47, 54] and has been demonstrated to improve implant stability and survival [8, 45, 54].

Research indicates that an optimal cement penetration depth of 3–4 mm provides maximum fixation at the cement–bone interface [14, 15, 26, 46]. Several factors contribute to improved cement penetration, including uniform bone density with adequate drill‐hole interdigitation, minimized intraoperative bleeding [16, 30, 40], the use of pulsed lavage [5, 27, 39, 55], and the absence of conditions such as sclerosis [13, 27]. Additional considerations, such as removing bone debris from cancellous bone [30, 32] and eliminating blood at the cement–bone interface [55], are also essential for optimizing cement penetration.

A tourniquet is a common practice during TKA. According to a survey conducted among members of the *American Association of Hip and Knee Surgeons*, 95% reported utilizing a tourniquet during TKA [7]. One notable advantage of tourniquet application in cemented TKA is the enhanced penetration of bone cement, attributed to reduced cancellous bone bleeding and the removal of clot debris during the cementing process [46]. Additionally, creating a bloodless surgical field through a tourniquet application improves visualization, facilitating the cementing quality [50].

Our analysis indicates that using a tourniquet significantly affects cement penetration at the tibial site, consistent with those reported in this study and two previous meta‐analyses [36, 49].

Sun et al. [49] found that increased tibial cement mantle thickness was primarily observed in Zone 3 on the AP view, with no significant increase in Zones 1, 2 and 4 on the AP view and Zones 1 and 2 on the lateral view. Similarly, the Subgroup analysis in our study revealed significant variability across different zones. It showed the ‘AP a (1,2)’ zone demonstrated a moderate effect, suggesting a more pronounced benefit of the intervention in this group, while other zones, such as ‘Lat2 (Pos)’ and ‘AP b (3,4)’, showed either negligible or no significant effects.

It has been hypothesized that improved visualization provided by a tourniquet could reduce the duration of surgery [11, 19, 20]. Nevertheless, our review showed that using a tourniquet does not influence surgical duration. This conclusion contrasts with the findings of the most recent meta‐analysis on this topic [3].

Numerous studies have demonstrated that using a tourniquet in TKA can increase postoperative pain [1, 33]. Prolonged tourniquet application has been found to exacerbate hypoxia in the surrounding soft tissues, leading to severe inflammation and muscle damage, which may intensify postoperative pain [41]. Additionally, congestive swelling of the soft tissue capsule can contribute to increased hidden blood loss and cause direct damage to nerve structures and soft tissues [1, 34, 52, 60]. Conversely, reducing the inflation pressure and duration of tourniquet application has decreased postoperative pain and associated complications [38, 56]. However, these findings contrast with the results of our study. In our analysis, pain was assessed using the VAS, and no significant differences in postoperative pain were observed between the two study groups.

The primary rationale for employing a tourniquet during TKA has been to minimize blood loss. Theoretically, premature tourniquet release can substantially increase visible and hidden blood loss. However, existing studies present conflicting evidence regarding the effectiveness of tourniquet use in reducing blood loss. Some researchers suggest that tourniquet application may paradoxically increase blood loss due to post‐ischaemic fibrinolysis [2, 29]. Factors such as hyperperfusion after deflation and activation of fibrinolytic enzymes may contribute to heightened postoperative blood loss [42]. Interestingly, some studies have found that fibrinolysis remains active for a brief period (approximately 30 min), which may not suffice as the primary cause of significant postoperative bleeding [9]. Variations in cuff inflation pressures (ranging from 250 to 380 mmHg) and inconsistencies in statistical methodologies for measuring blood loss across studies could also contribute to these conflicting findings [3]. Our study evaluated blood loss by examining the need for blood transfusions and Hb level changes, revealing that patients in the tourniquet group had a 52% lower likelihood of requiring a blood transfusion.

The observed phenomenon, where Hb levels did not significantly differ between groups, yet the tourniquet group exhibited a lower blood transfusion rate, can be attributed to several factors. First, tourniquet application during surgery effectively reduces intraoperative blood loss by restricting blood flow to the operative field, which may not be immediately reflected in postoperative haemoglobin measurements due to fluid shifts and hemodilution [4]. Second, transfusion decisions are often influenced by clinical judgement and visible blood loss rather than solely on Hb levels; thus, reduced intraoperative bleeding in the tourniquet group could lead to fewer transfusions despite similar Hb readings [10]. Additionally, variations in transfusion protocols and patient responses to blood loss can contribute to this discrepancy. Therefore, while Hb levels provide a snapshot of a patient's blood status, they may not fully capture the dynamic and multifactorial nature of blood loss and transfusion requirements during and after surgery.

This meta‐analysis is subject to several limitations. First, heterogeneity was observed across the included studies (e.g., *I* ^2^ values ranging from 75.2% to 92.6%), indicating variability in study designs, populations and interventions. Differences in prosthesis brands, bone cement brands, cementing technique and tourniquet protocols, such as the duration, pressure applied and timing of release, may have significantly influenced outcomes and complicated direct comparisons between studies. Additionally, variability in reporting outcomes, including functional measures, intraoperative blood loss and postoperative pain, highlights a lack of standardization, making it challenging to draw definitive conclusions. Another limitation is the potential for selection bias, as not all studies may have been included due to language restrictions, incomplete data, or publication bias favouring studies with significant findings. The follow‐up durations were also inconsistent, with many studies potentially underreporting long‐term complications or benefits associated with tourniquet use. Finally, patient‐related factors such as age, BMI, comorbidities and surgical techniques (e.g., minimally invasive vs. traditional TKA approaches) were often inadequately controlled, which may have introduced confounding variables. Future research should address these issues by employing standardized methodologies, uniform outcome measures, and robust study designs to improve the reliability and applicability of the findings.

## CONCLUSION

This meta‐analysis investigated the impact of tourniquet use during TKA, primarily focusing on cement penetration. The findings indicate that tourniquet application may enhance cement penetration, which could play a critical role in achieving mechanical interlock and implant stability, given the non‐adhesive nature of bone cement. However, the relationship between tourniquet use, cement penetration, and long‐term implant fixation warrants further direct investigation. No significant differences were found in surgical time or postoperative pain. Although a lower transfusion rate was noted, the outcomes of this review should be interpreted with caution given the high heterogeneity among studies and the influence of other critical factors such as cement type, cementing technique, surgical protocols, and limited reporting.

## AUTHOR CONTRIBUTIONS

Alireza Mirahmadi, Ara Nazarian, Shayan Amiri, Hamed Tayyebi and Mengnai Li designed the study. Ava Parvandi, Donya Rezazadeh Eidgahi and Amirsina Shaker Dorabad did the search and screening. Alireza Mirahmadi, Ava Parvandi, Amirsina Shaker Dorabad and Mahdi Mohammaditabar did the quality assessments. Ara Nazarian, Alireza Mirahmadi, Mengnai Li, Shayan Amiri and Hamed Tayyebi resolved conflicts in each step. Alireza Mirahmadi and Ava Parvandi drafted the manuscript and tables. All authors read and approved the final.

## CONFLICT OF INTEREST STATEMENT

The authors declare no conflicts of interest.

## ETHICS STATEMENT

This systematic review and meta‐analysis adhered to the PRISMA guidelines (Preferred Reporting Items for Systematic Reviews and Meta‐Analyses) and was registered in the PROSPERO database under the registration code CRD42024627837.

## Data Availability

All data generated or analyzed during this study are included in this published article.

## APPENDIX A

See Table A1.

**Table A1:** Search strategy for all databases is available.

PubMed	Embase	Scopus	WOS
(‘Bone Cements’[MeSH] OR ‘bone cement’ OR ‘bone glue*’ OR ‘bone paste*’)	(‘bone cement’/exp OR ‘bone cement’ OR ‘bone glue* OR ‘bone paste*’)	TITLE‐ABS‐KEY(‘bone cement’ OR ‘bone glue*’ OR ‘bone paste*’)	TS = (‘bone cement’ OR ‘bone glue* OR ‘bone paste*’)
AND			
(‘Tourniquets’[MeSH] OR tourniquet)	(‘tourniquet’/exp OR tourniquet)	TITLE‐ABS‐KEY(tourniquet)	TS = (tourniquet)
AND			
(‘Arthroplasty, Replacement, Knee’[MeSH] OR ‘total knee arthroplasty’ OR ‘TKA’ OR ‘knee replacement’ OR ‘partial knee replacement’ OR ‘unicompartmental knee arthroplasty’ OR ‘unicondylar knee arthroplasty’)	(‘knee arthroplasty’/exp OR ‘knee replacement’ OR ‘total knee arthroplasty’ OR ‘unicompartmental knee arthroplasty’ OR ‘partial knee replacement’ OR ‘unicondylar knee arthroplasty’)	TITLE‐ABS‐KEY(‘total knee arthroplasty’ OR ‘knee replacement’ OR ‘partial knee replacement’ OR ‘unicompartmental knee arthroplasty’ OR ‘unicondylar knee arthroplasty’)	TS = (‘knee replacement’ OR ‘total knee arthroplasty’ OR ‘TKA’ OR ‘partial knee replacement’ OR ‘unicompartmental knee arthroplasty’ OR ‘unicondylar knee arthroplasty’)
AND			
(‘Haemorrhage, Surgical’[MeSH] OR ‘blood loss, surgical’ OR hemorrhage OR bleeding OR ‘cement penetration’)	(‘surgical hemorrhage’/exp OR ‘blood loss’/exp OR hemorrhage OR bleeding OR ‘cement penetration’)	TITLE‐ABS‐KEY(‘surgical hemorrhage’ OR ‘blood loss’ OR hemorrhage OR bleeding OR ‘cement penetration’)	TS = (‘surgical hemorrhage’ OR ‘blood loss’ OR hemorrhage OR bleeding OR ‘cement penetration’)

## References

[1] Abdel‐Salam A , Eyres KS . Effects of tourniquet during total knee arthroplasty. A prospective randomised study. J Bone Joint Surg Br. 1995;77(2):250–253. 7706340

[2] Aglietti P , Baldini A , Vena LM , Abbate R , Fedi S , Falciani M . Effect of tourniquet use on activation of coagulation in total knee replacement. Clin Orthop Relat Res. 2000;371:169–177. 10.1097/00003086-200002000-00021 10693564

[3] Ahmed I , Chawla A , Underwood M , Price AJ , Metcalfe A , Hutchinson CE , et al. Time to reconsider the routine use of tourniquets in total knee arthroplasty surgery. Bone Joint J. 2021;103–B(5):830–839. 10.1302/0301-620X.103B.BJJ-2020-1926.R1 PMC8091001 33683139

[4] Arafah OM , Alotaibi AM , Alsalloum AM , Alotaibi HM . Safety and blood loss associated with tourniquets in total knee arthroplasty. Cureus. 2021;13(8):16875. 10.7759/cureus.16875 PMC8412002 34513450

[5] Baumann C , Baumann J , Bozynski C , Stoker A , Stannard J , Cook J . Comparison of techniques for preimplantation treatment of osteochondral allograft bone. J Knee Surg. 2019;32(1):097–104. 10.1055/s-0038-1636834 29514363

[6] Bauze A , Costi J , Stavrou P , Rankin W , Hearn T , Krishnan J , et al. Cement penetration and stiffness of the cement‐bone composite in the proximal tibia in a porcine model. J Orthop Surg. 2004;12(2):194–198. 10.1177/230949900401200211 15621906

[7] Berry DJ , Bozic KJ . Current practice patterns in primary hip and knee arthroplasty among members of the American Association of Hip and Knee Surgeons. J Arthroplasty. 2010;25(6 Suppl):2–4. 20580196 10.1016/j.arth.2010.04.033

[8] Bert JM , McShane M . Is it necessary to cement the tibial stem in cemented total knee arthroplasty? Clin Orthop Relat Res. 1998;356:73–78. 10.1097/00003086-199811000-00012 9917670

[9] Blanié A , Bellamy L , Rhayem Y , Flaujac C , Samama CM , Fontenay M , et al. Duration of postoperative fibrinolysis after total hip or knee replacement: a laboratory follow‐up study. Thromb Res. 2013;131(1):6. 10.1016/j.thromres.2012.11.006 23195143

[10] Burkart BC , Bourne RB , Rorabeck CH , Kirk PG , Nott L . The efficacy of tourniquet release in blood conservation after total knee arthroplasty. Clin Orthop Relat Res. 1994;299:147–152. 8119009

[11] Cai DF , Fan QH , Zhong HH , Peng S , Song H . The effects of tourniquet use on blood loss in primary total knee arthroplasty for patients with osteoarthritis: a meta‐analysis. J Orthop Surg. 2019;14(1):348. 10.1186/s13018-019-1422-4 PMC6839231 31703706

[12] Cawley DT , Kelly N , McGarry JP , Shannon FJ . Cementing techniques for the tibial component in primary total knee replacement. Bone Joint J. 2013;95–B(3):295–300. 10.1302/0301-620X.95B3.29586 23450010

[13] Dennis DA , Kittelson AJ , Yang CC , Miner TM , Kim RH , Stevens‐Lapsley JE . Does tourniquet use in TKA affect recovery of lower extremity strength and function? A randomized trial. Clin Orthop Relat Res. 2016;474(1):69–77. 26100254 10.1007/s11999-015-4393-8 PMC4686529

[14] Ejaz A , Laursen AC , Jakobsen T , Rasmussen S , Nielsen PT , Laursen MB . Absence of a tourniquet does not affect fixation of cemented TKA: A randomized RSA study of 70 patients. J Arthroplasty. 2015;30(12):2128–2132. 26162514 10.1016/j.arth.2015.05.058

[15] Ejaz A , Laursen AC , Kappel A , Laursen MB , Jakobsen T , Rasmussen S , et al. Faster recovery without the use of a tourniquet in total knee arthroplasty. Acta Orthop. 2014;85(4):422–426. 24954487 10.3109/17453674.2014.931197 PMC4105775

[16] Fehring TK , Odum S , Griffin WL , Mason JB , Nadaud M . Early failures in total knee arthroplasty. Clin Orthop Relat Res. 2001;392:315–318. 10.1097/00003086-200111000-00041 11716402

[17] Gao PLH , Bai Z , Zhang P . Effect of not using tourniquet during TKA on early prosthesis stability and knee function. Hin J Bone Joint Injury. 2019;34(12): 1294–1296.

[18] Gapinski ZA , Yee EJ , Kraus KR , Deckard ER , Meneghini RM . The effect of tourniquet use and sterile carbon dioxide gas bone preparation on cement penetration in primary total knee arthroplasty. J Arthroplasty. 2019;34(8):1634–1639. 31010776 10.1016/j.arth.2019.03.050

[19] Zhang W , Li N , Chen S , Tan Y , Al‐Aidaros M , Chen L , et al. The effects of a tourniquet used in total knee arthroplasty: a meta‐analysis. J Orthop Surg. 2014;9(1):13. 10.1186/1749-799X-9-13 PMC3973857 24602486

[20] Goel R , Rondon AJ , Sydnor K , Blevins K , O'Malley M , Purtill JJ , et al. Tourniquet use does not affect functional outcomes or pain after total knee arthroplasty: a prospective, double‐blinded, randomized controlled trial. J Bone Joint Surg Am. 2019;101(20):1821–1828. 31626006 10.2106/JBJS.19.00146

[21] Hegde V , Bracey DN , Johnson RM , Dennis DA , Jennings JM . Tourniquet use improves cement penetration and reduces radiolucent line progression at 5 years after total knee arthroplasty. J Arthroplasty. 2021;36(7s):209. 10.1016/j.arth.2020.12.048 33500203

[22] Herndon CL , Grosso MJ , Sarpong NO , Shah RP , Geller JA , Cooper HJ . Tibial cement mantle thickness is not affected by tourniquetless total knee arthroplasty when performed with tranexamic acid. Knee Surg Sports Traumatol Arthrosc. 2020;28(5):1526–1531. 31190247 10.1007/s00167-019-05559-3

[23] Hofmann AA , Goldberg TD , Tanner AM , Cook TM . Surface cementation of stemmed tibial components in primary total knee arthroplasty: minimum 5‐year follow‐up. J Arthroplasty. 2006;21(3):353–357. 16627142 10.1016/j.arth.2005.06.012

[24] Inui H , Yamagami R , Kono K , Kawaguchi K . What are the causes of failure after total knee arthroplasty? J Jt Surg Res. 2023;1(1):32–40.

[25] Janssen D , Mann KA , Verdonschot N . Micro‐mechanical modeling of the cement‐bone interface: the effect of friction, morphology and material properties on the micromechanical response. J Biomech. 2008;41(15):3158–3163. 18848699 10.1016/j.jbiomech.2008.08.020 PMC2613656

[26] Jawhar A , Stetzelberger V , Kollowa K , Obertacke U . Tourniquet application does not affect the periprosthetic bone cement penetration in total knee arthroplasty. Knee Surg Sports Traumatol Arthrosc. 2019;27(7):2071–2081. 30539303 10.1007/s00167-018-5330-7

[27] Kheir MM , Ziemba‐Davis M , Dilley JE , Hood Jr, MJ , Meneghini RM . Tourniquetless total knee arthroplasty with modern perioperative protocols decreases pain and opioid consumption in women. J Arthroplasty. 2018;33(11):3455–3459. 30075878 10.1016/j.arth.2018.06.038

[28] Kizilkurt T , Bayram S , Ekinci M , Ayik Ö , Ergin ÖN , Öztürk İ . Comparing the effect of tourniquet and tranexamic acid on the tibial cement mantle thickness in total knee arthroplasty. Eur J Orthop Surg Traumatol. 2022;32(2):263–268. 33811527 10.1007/s00590-021-02961-x

[29] Klenerman L , Chakrabarti R , Mackie I , Brozovic M , Stirling Y . Changes in haemostatic system after application of a tourniquet. Lancet. 1977;1(8019):970–972. 67466 10.1016/s0140-6736(77)92276-0

[30] Kurtz S , Ong K , Lau E , Mowat F , Halpern M . Projections of primary and revision hip and knee arthroplasty in the United States from 2005 to 2030. J Bone Joint Surg Am. 2007;89(4):780–785. 17403800 10.2106/JBJS.F.00222

[31] Lai Y , Xu H , Su Q , Wan X , Yuan M , Zhou Z . Effect of tourniquet use on blood loss, pain, functional recovery, and complications in robot‐assisted total knee arthroplasty: a prospective, double‐blinded, randomized controlled trial. J Orthop Surg. 2022;17(1):118. 10.1186/s13018-022-02992-y PMC8862211 35189911

[32] Lange JK , Lee YY , Spiro SK , Haas SB . Satisfaction rates and quality of life changes following total knee arthroplasty in age‐differentiated cohorts. J Arthroplasty. 2018;33(5):1373–1378. 29395722 10.1016/j.arth.2017.12.031

[33] Ledin H , Aspenberg P , Good L . Tourniquet use in total knee replacement does not improve fixation, but appears to reduce final range of motion. Acta Orthop. 2012;83(5):499–503. 22974220 10.3109/17453674.2012.727078 PMC3488177

[34] Li B , Wen Y , Wu H , Qian Q , Lin X , Zhao H . The effect of tourniquet use on hidden blood loss in total knee arthroplasty. Int Orthop. 2009;33(5):1263–1268. 18751703 10.1007/s00264-008-0647-3 PMC2899119

[35] Li X , Liu J , Wang H , Ding Y . Controlled hypotension technology can improve patient recovery in the early postoperative period after total knee arthroplasty: a prospective, randomized controlled clinical study. Jt Dis Relat Surg. 2023;35(1):36–44. 38108164 10.52312/jdrs.2023.1379 PMC10746890

[36] Lu C , Song M , Chen J , Li C , Lin W , Ye G , et al. Does tourniquet use affect the periprosthetic bone cement penetration in total knee arthroplasty? A meta‐analysis. J Orthop Surg. 2020;15(1):602. 10.1186/s13018-020-02106-6 PMC7730759 33308270

[37] Maistrelli GL , Antonelli L , Fornasier V , Mahomed N . Cement penetration with pulsed lavage versus syringe irrigation in total knee arthroplasty. Clin Orthop Relat Res. 1995;312:261–265. 7634612

[38] Manén Berga F , Novellas Canosa M , Anglès Crespo F , Bernal Dzekonski J . Effect of ischemic tourniquet pressure on the intensity of postoperative pain. Rev Esp Anestesiol Reanim. 2002;49(3):131–135. 12136454

[39] Meyer MA , McCarthy MA , Gitelis ME , Poland SG , Urita A , Chubinskaya S , et al. Effectiveness of lavage techniques in removing immunogenic elements from osteochondral allografts. Cartilage. 2017;8(4):369–373. 28934881 10.1177/1947603516681132 PMC5613898

[40] Molt M , Harsten A , Toksvig‐Larsen S . The effect of tourniquet use on fixation quality in cemented total knee arthroplasty a prospective randomized clinical controlled RSA trial. Knee. 2014;21(2):396–401. 24238650 10.1016/j.knee.2013.10.008

[41] Olivecrona C , Lapidus LJ , Benson L , Blomfeldt R . Tourniquet time affects postoperative complications after knee arthroplasty. Int Orthop. 2013;37(5):827–832. 23417522 10.1007/s00264-013-1826-4 PMC3631475

[42] Orpen NM , Little C , Walker G , Crawfurd EJP . Tranexamic acid reduces early post‐operative blood loss after total knee arthroplasty: a prospective randomised controlled trial of 29 patients. Knee. 2006;13(2):106–110. 16487712 10.1016/j.knee.2005.11.001

[43] Ozkunt O , Sariyilmaz K , Gemalmaz HC , Dikici F . The effect of tourniquet usage on cement penetration in total knee arthroplasty: a prospective randomized study of 3 methods. Medicine. 2018;97(4):e9668. 29369184 10.1097/MD.0000000000009668 PMC5794368

[44] Page MJ , McKenzie JE , Bossuyt PM , et al. The PRISMA 2020 statement: an updated guideline for reporting systematic reviews. BMJ. 2021;372:n71. 33782057 10.1136/bmj.n71 PMC8005924

[45] Peters CL , Craig MA , Mohr RA , Bachus KN . Tibial component fixation with cement: full‐ versus surface‐cementation techniques. Clin Orthop Relat Res. 2003;409:158–168. 10.1097/01.blo.0000058638.94987.20 12671498

[46] Pfitzner T , von Roth P , Voerkelius N , Mayr H , Perka C , Hube R . Influence of the tourniquet on tibial cement mantle thickness in primary total knee arthroplasty. Knee Surg Sports Traumatol Arthrosc. 2016;24(1):96–101. 25248311 10.1007/s00167-014-3341-6

[47] Schlegel UJ , Bishop NE , Püschel K , Morlock MM , Nagel K . Comparison of different cement application techniques for tibial component fixation in TKA. Int Orthop. 2015;39(1):47–54. 25082179 10.1007/s00264-014-2468-x

[48] Sharkey PF , Hozack WJ , Rothman RH , Shastri S , Jacoby SM . Insall Award paper. Why are total knee arthroplasties failing today? Clin Orthop Relat Res. 2002;404:7–13. 10.1097/00003086-200211000-00003 12439231

[49] Sun C , Yang X , Zhang X , Ma Q , Yu P , Cai X , et al. The impact of tourniquet on tibial bone cement penetration in different zones in primary total knee arthroplasty: a meta‐analysis. J Orthop Surg. 2021;16(1):198. 10.1186/s13018-021-02345-1 PMC7968365 33731155

[50] Tai TW , Lin CJ , Jou IM , Chang CW , Lai KA , Yang CY . Tourniquet use in total knee arthroplasty: a meta‐analysis. Knee Surg Sports Traumatol Arthrosc. 2011;19(7):1121–1130. 21161177 10.1007/s00167-010-1342-7 PMC3116117

[51] Touzopoulos P , Ververidis A , Mpogiatzis C , Chatzigiannakis A , Drosos GI . The use of tourniquet may influence the cement mantle thickness under the tibial implant during total knee arthroplasty. Eur J Orthop Surg Traumatol. 2019;29(4):869–875. 30617921 10.1007/s00590-019-02369-8

[52] Vandenbussche E , Duranthon LD , Couturier M , Pidhorz L , Augereau B . The effect of tourniquet use in total knee arthroplasty. Int Orthop. 2002;26(5):306–309. 12378360 10.1007/s00264-002-0360-6 PMC3621001

[53] Vertullo CJ , Nagarajan M . Is cement penetration in TKR reduced by not using a tourniquet during cementation? A single blinded, randomized trial. J Orthop Surg. 2017;25(1):2309499016684323. 10.1177/2309499016684323 28139192

[54] Walker PS , Soudry M , Ewald FC , McVickar H . Control of cement penetration in total knee arthroplasty. Clin Orthop Relat Res. 1984;185:155–164. 6705374

[55] Weinstein AM , Rome BN , Reichmann WM , Collins JE , Burbine SA , Thornhill TS , et al. Estimating the burden of total knee replacement in the United States. J Bone Jt Surg. 2013;95(5):385–392. 10.2106/JBJS.L.00206 PMC3748969 23344005

[56] Worland RL , Arredondo J , Angles F , Lopez‐Jimenez F , Jessup DE . Thigh pain following tourniquet application in simultaneous bilateral total knee replacement arthroplasty. J Arthroplasty. 1997;12(8):848–852. 9458249 10.1016/s0883-5403(97)90153-4

[57] Xie X , Yue C , Huang Z , Kang PD , Zhou ZK , Yang J , et al. Total knee arthroplasty with or without tourniquet: a randomized controlled trial. Orthop J China. 2017;25(17):1572–1576.

[58] Yang J , Wei L , Zhang J , Huang X . The effect of the tourniquet on cement mantle thickness in total knee arthroplasty. n.d. 10.3969/j.issn.1671-8348.2017.06.020.

[59] Yi Z , Yan L , Haibo S , Yuangang W , Mingyang L , Yuan L , et al. Effects of tourniquet use on clinical outcomes and cement penetration in TKA when tranexamic acid administrated: a randomized controlled trial. BMC Musculoskelet Disord. 2021;22(1):126. 33517881 10.1186/s12891-021-03968-5 PMC7847577

[60] Zhang Y , Li L , Wang J , Li Z , Shi Z . Do patients benefit from tourniquet in arthroscopic surgeries of the knee? Knee Surg Sports Traumatol Arthrosc. 2013;21(5):1125–1130. 22699853 10.1007/s00167-012-2094-3

[61] Zhou J , Dou W , Oouyang H , Chu W . The effect of not using tourniquet in total knee arthroplasty. Chin Rural Med. 2018;25(23):10–11.

